# External Use of Ipecac

**Published:** 1853-10

**Authors:** 


### External use of Ipecac.

The *Bulletin de Therapeutique*, (Revue Medico-Chirurgicale, 1853,) contains a paper by Dr. Detroux, on the external use of ipecacuanha, as a valuable counter-irritant. The pommade of ipecac possesses the same advantages, says Dr. D., as the ointment of tartar emetic, provided that it be incorporated with some fatty body, and be then rubbed over the surface of the part sought to be irritated, for a few minutes. Thus employed, it will speedily develope a characteristic exanthema in the shape of small papular elevations of an intense rose color, very numerous, and often confluent. Soon they assume the form of genuine pustules, but small, depressed in the centre—umbilicated—inclined to suppurate, and soon disappearing if the ointment be not renewed. It causes much less pain, and leaves behind smaller cicatrices than the tartar emetic ointment. He uses the following formulæ:

℞ Pulvis Ipecac, 1 part,

Ol. Olivæ, 1 part,

Axungia, 2 parts, M

Fit. ungt.

It is well adapted to children and delicate nervous subjects, on whose system the tartar emetic would be likely to produce evil effects.

